# Monte Carlo simulations of optical propagation in human skin using experimentally measured laser parameters

**DOI:** 10.1007/s10103-026-04911-7

**Published:** 2026-06-09

**Authors:** Carlos Eduardo Girasol, Raíssa Mendonça Quaranta Lobão, Murilo Sanches Sampaio, Luismar Barbosa Cruz-Junior, Rinaldo Roberto de Jesus Guirro, Luciano Bachmann

**Affiliations:** 1 Department of Physics, Universidade de São Paulo, Ribeirão Preto, Brazil; 2 Universidade do Estado de Santa Catarina (Udesc), Florianópolis, Brazil; 3 Department of Health Sciences, Universidade de São Paulo, Ribeirão Preto, Brazil

**Keywords:** Tissue Optical Parameters, Low-Level Light Therapy, Light-emitting Diode, Photobiomodulation, Monte Carlo Method, Physical Therapy

## Abstract

To simulate light propagation in human skin irradiated with laser sources emitting at 660 nm, 830 nm, and 904 nm, using different beam diameters and divergences, in order to characterize the internal fluence rate distribution profiles within the tissue. Monte Carlo simulations were performed using the GPU-accelerated MCX platform. Optical properties (absorption and reduced scattering coefficients) were experimentally obtained from human skin samples and incorporated into a three-dimensional voxel-based model. Laser parameters, including output power, beam radius, and divergence, were experimentally characterized and used as input for simulations. The effects of wavelength, beam divergence, and source radius on axial and spatial fluence rate distribution were quantitatively evaluated under controlled power conditions. All wavelengths exhibited exponential attenuation with comparable depth-dependent behavior within the first millimeters of tissue. Beam radius significantly modulated surface peak fluence rate and central-axis magnitude at depth under constant total power. In contrast, real divergence values typical of commercial devices produced minimal changes in axial fluence under contact-mode conditions, whereas large theoretical divergence angles markedly reduced fluence retention and increased lateral spread. Among the investigated variables, beam radius exerted the strongest influence on subsurface fluence magnitude under constant power, whereas wavelengths within the therapeutic window (660–904 nm) showed comparable attenuation behavior in superficial tissue. Realistic divergence values typical of commercial devices minimally affected axial fluence under contact-mode conditions, while larger angular spreads significantly reduced central-axis fluence retention.

## Introduction

The optical properties of human skin are important for a wide range of therapeutic and diagnostic applications, including photobiomodulation therapy (PBMT), photodynamic therapy (PDT), and optical diagnostic tools such as spectroscopy, particularly in dermatology and physiotherapy [1, 2]. The interaction between optical radiation and biological tissues is governed by photon transport, spatial energy deposition, and ultimately the biological response induced by these processes [3]. Although light-based therapies are increasingly employed in clinical practice, uncertainties persist regarding subsurface photon propagation and internal fluence rate distribution. A precise understanding of these mechanisms is essential to ensure accurate dosimetry, reproducibility of outcomes, and effective targeting of deeper tissues.

The energy density delivered to biological tissues depends not only on intrinsic optical properties but also on the physical characteristics of the irradiation source. The internal power density distribution within tissue, which determines treatment accuracy [4], is influenced by parameters such as wavelength, output power, pulse energy, beam diameter, and divergence, in addition to tissue optical properties. These properties are associated with light–matter interactions, including reflection, scattering, absorption, and refraction [5–7]. When incident photons interact with cellular structures and molecules, they may be absorbed and deposit energy, thereby inducing biological effects. While absorption is the primary mechanism responsible for therapeutic effects in PBMT and PDT, scattering plays a dominant role in determining penetration depth and spatial fluence distribution in skin. In this context, the key optical properties that characterize biological tissues include the absorption coefficient $\:\left({\mu\:}_{a}\right)$, scattering coefficient $\:\left({\mu\:}_{s}\right)$, anisotropy factor $\:\left(g\right)$, and refractive index $\:\left(n\right)$ [7, 8].

Experimental approaches to assessing light propagation in biological tissues are often constrained by ethical concerns, sample variability, and technical challenges in measuring subsurface light distribution. Consequently, computational modeling has become an essential tool for investigating photon transport in turbid media such as human skin [9, 10]. Among available approaches, Monte Carlo (MC) simulations are considered the gold standard due to their ability to stochastically model photon–tissue interactions based on radiative transfer theory [11].

Given the widespread clinical use of therapeutic light sources, understanding the internal fluence rate distribution profile generated by laser devices with realistic geometric and divergence parameters is fundamental for improving dosimetric precision. By characterizing this profile, laser-based procedures can be performed with greater precision, ensuring that the target tissue receives the intended fluence of optical radiation. However, many computational studies rely on idealized beam assumptions that may not reflect the actual geometric and divergence characteristics of commercial therapeutic sources. A quantitative optical dosimetry analysis incorporating experimentally measured laser parameters is therefore necessary to bridge this gap and support the development of standardized clinical protocols.

The wavelengths selected in this study (660, 830, and 904 nm) represent some of the most commonly used laser sources in clinical photobiomodulation practice and span relevant regions of the optical therapeutic window. The 660 nm wavelength is frequently used for superficial targets, whereas 830 nm and 904 nm are commonly employed for deeper tissue applications. Their inclusion also enables comparison of wavelength-dependent fluence behavior using clinically realistic devices. Therefore, the objective of this study was to simulate light propagation in human skin irradiated with laser sources emitting at 660 nm, 830 nm, and 904 nm, using different beam diameters and divergences, in order to characterize the internal fluence rate distribution profiles within the tissue.

## Materials and methods

### Design

This study employed a Monte Carlo computational approach to analyze light propagation in human skin irradiated with laser sources emitting at 660 nm, 830 nm, and 904 nm, using varying beam diameters and divergence angles. The simulations were designed to evaluate the interaction between experimentally measured source parameters and the optical properties of the irradiated tissue.

Photon transport within a three-dimensional (3D) tissue model was simulated using a stochastic framework based on radiative transfer theory [12]. In this approach, photon packets are launched into the medium and undergo probabilistic absorption and scattering events governed by the tissue’s optical parameters. The MC method enables accurate modeling of photon migration in turbid biological media and has been widely validated for light–tissue interaction studies.

The simulations were implemented using the GPU-accelerated Monte Carlo eXtreme (MCX) platform developed by Fang and Boas [11], allowing efficient computation of large photon populations and high-resolution spatial fluence distributions.

### Light sources

Three commercially available therapeutic laser devices emitting at 660 nm, 830 nm, and 904 nm were experimentally characterized for use as simulation inputs. These wavelengths are among the most commonly used in clinical photobiomodulation practice. The average power of each device was measured using a calibrated PowerMax-USB sensor (Coherent, USA) connected to dedicated acquisition software. The sensor had been factory-calibrated within the previous 12 months, ensuring a measurement uncertainty between 1% and 1.5%, according to manufacturer specifications. During measurements, the laser probes were mechanically stabilized and positioned perpendicular to the sensor surface to ensure reproducible alignment.

Beam diameter and spatial power distribution were determined using radiation-sensitive detectors appropriate for each spectral range (LM-2 VIS for visible wavelengths and LM-2 NIR for near-infrared; Coherent, USA), coupled to a FieldMaxII TOP power meter via a 0.25 mm diameter optical fiber. The detection system was mounted on a high-precision XYZ translation stage (0.005 mm resolution), enabling systematic mapping of the beam profile across orthogonal axes.

For each source, transverse power scans were performed at the contact position (Z = 0 mm) and at increasing distances from the aperture (3, 6, 9, 12, and 15 mm). Gaussian fitting was applied to the measured intensity profiles to determine the effective beam radius. In addition, full cross-sectional mapping enabled reconstruction of the three-dimensional spatial power distribution. As the intrinsic beam structure remains geometrically preserved along propagation, detailed 3D mapping was not repeated at each Z-position.

A comprehensive description of the experimental characterization protocol has been previously reported by Girasol et al. [13]. The measured parameters used as input for the simulations are summarized in Table 1.

**Table 1:** Representation of the physical parameters used during photobiomodulation therapy

Parameters	Source 1	Source 2	Source 3
Wavelength	660 nm	830 nm	904 nm
Frequency	Continuous wave	Continuous wave	Continuous wave
Spot area (cm²)	0.07 cm^2^	0.12 cm^2^	0.13 cm^2^
Power output	40 mW	30 mW	70 mW
Power density	0.57 W/cm^2^	0.25 W/cm^2^	0.54 W/cm^2^
Output diameter	0.1636 mm	0.1576 mm	0.1768 mm
Divergence	0.083 rad	0.112 rad	0.089 rad

### Monte Carlo simulations

Monte Carlo simulations were performed using the GPU-accelerated MCX framework through its Python interface (PyMCX) [11, 14]. The MCX packages are available as open-access repositories on GitHub. Access to the MCX control codes can be obtained through the official website or directly via the provided link [15]. The simulation workflow consisted of defining the optical properties of the tissue, the geometric configuration of the irradiation source, and the computational domain parameters.

Photon packets were launched perpendicularly onto the tissue surface with an initial weight proportional to the total emitted energy. Photon propagation within the three-dimensional voxel-based domain was modeled according to radiative transfer principles, with absorption and scattering events governed by the specified absorption and reduced scattering coefficient, anisotropy factor, and refractive index. The Henyey–Greenstein phase function was applied to describe scattering angular distributions. At each interaction, photon weight was reduced proportionally to local absorption, while scattering events altered propagation direction. Photon packets were terminated when their weight fell below a predefined threshold or when they exited the simulation domain.

The initial computational domain was defined as a 30 × 30 × 30 mm³ volume, discretized into isotropic voxels with a side length of 0.025 mm. Considering the optical properties used in this study, the transport mean free path is approximately 0.5 mm, corresponding to approximately 20 voxels, ensuring adequate spatial resolution to capture near-surface fluence gradients. Domain dimensions were adjusted when necessary to ensure complete inclusion of the beam expansion and optical penetration depth, while maintaining computational efficiency. For each simulation condition, a sufficiently large number of photon packets (≥ 10⁸ photons) was used to ensure convergence and statistical stability of the fluence distribution maps. Each simulation was repeated five times using different random seeds, and the reported results correspond to the average of these runs. The relative variation across simulations was approximately 1%, indicating low statistical uncertainty.

### Simulation of laser irradiation

The simulated clinical configuration consisted of a laser source, applied in contact mode, defined by its measured beam diameter (ϕ input), wavelength (λ), and divergence angle (θ), directed to a skin sample of thickness (d_skin_), characterized by optical properties (µₐ, µₛ, g, and n). Beneath the skin lies the target tissue, the muscle, which was simplified as a homogeneous layer for simulation purposes. The refractive index mismatch between air (*n* = 1.0) and tissue (*n* = 1.37) was accounted for through Fresnel reflections at the tissue boundary, while assuming direct contact between the source and the tissue surface without an air gap.

Simulations were performed to determine the three-dimensional fluence distribution within the tissue and to quantify clinically relevant parameters, including transmitted power at the skin–muscle interface and the effective irradiated area at depth (A_muscle_). From these values, the power density delivered to the target tissue was calculated.

The influence of wavelength was evaluated by comparing simulations at 660 nm, 830 nm, and 904 nm, accounting for their respective optical properties. This analysis enabled assessment of penetration behavior and fluence distribution under clinically relevant spectral conditions.

To investigate geometric effects, beam diameter was systematically varied using experimentally measured values (Table 1), as well as simulated smaller and larger diameters. The resulting changes in transmitted energy and irradiated area at depth were quantified to determine the impact of source radius on subsurface power density.

Beam divergence was also assessed by comparing experimentally measured divergence values with a perpendicular collimated baseline (θ = 0º) and with increased divergence scenarios. This approach allowed evaluation of how angular spread influences axial energy concentration and depth-dependent fluence.

### Optical properties of skin

The optical properties used as input parameters for the simulations were experimentally obtained by the Photobiophysics Research Group in collaboration with the Laboratory of Physiotherapeutic Resources (LARF). The samples corresponded to abdominal skin from Caucasian individuals, and all procedures were approved by the Ethics Committee of the Clinics Hospital of the Medical School of Ribeirão Preto (CAAE: 90630218.2.0000.5440; approval number: 3.275.034) [6, 7].

The absorption and reduced scattering coefficients were determined using the Inverse Adding-Doubling (IAD) method based on diffuse reflectance and transmittance spectra acquired with a double integrating sphere system. The reduced scattering coefficient (µₛ′ = µₛ(1 − g)) was used as the primary scattering input parameter in the simulations, in conjunction with an anisotropy factor of g = 0.91.

Mean values ± standard deviation for µₐ and µₛ′ at 660 nm, 830 nm, and 904 nm are presented in Table 2. These experimentally derived parameters were directly incorporated into the Monte Carlo model without further adjustment.

**Table 2:** Average values of the absorption and reduced scattering coefficients of human skin samples used as input in the simulations

	660 nm	830 nm	904 nm
µa [mm − ^1^]	0.03316 ± 0.00670	0.01006 ± 0.00512	0.01038 ± 0.00622
µ’s [mm − ^1^]	1.88212 ± 0.15179	1.61533 ± 0.21205	1.37513 ± 0.22638

### Software

All simulations were performed using the Monte Carlo eXtreme (MCX) algorithm through its open-source Python interface (PyMCX 1.0), available at http://mcx.space [11]. The MCX framework leverages GPU-based parallel processing to efficiently simulate large photon populations within voxelized media.

Simulation parameters were configured via JSON input files, including domain dimensions, optical properties (µₐ, µₛ′, g, n), source geometry, divergence, and total photon number [16]. Execution and data processing were conducted using PyCharm.

Computations were performed on a workstation equipped with an Intel Core i5-13450HX processor, 16 GB RAM, and an NVIDIA RTX 3050 GPU (6 GB VRAM).

## Results

### Effect of wavelength on fluence decay

**Fig. 1:**
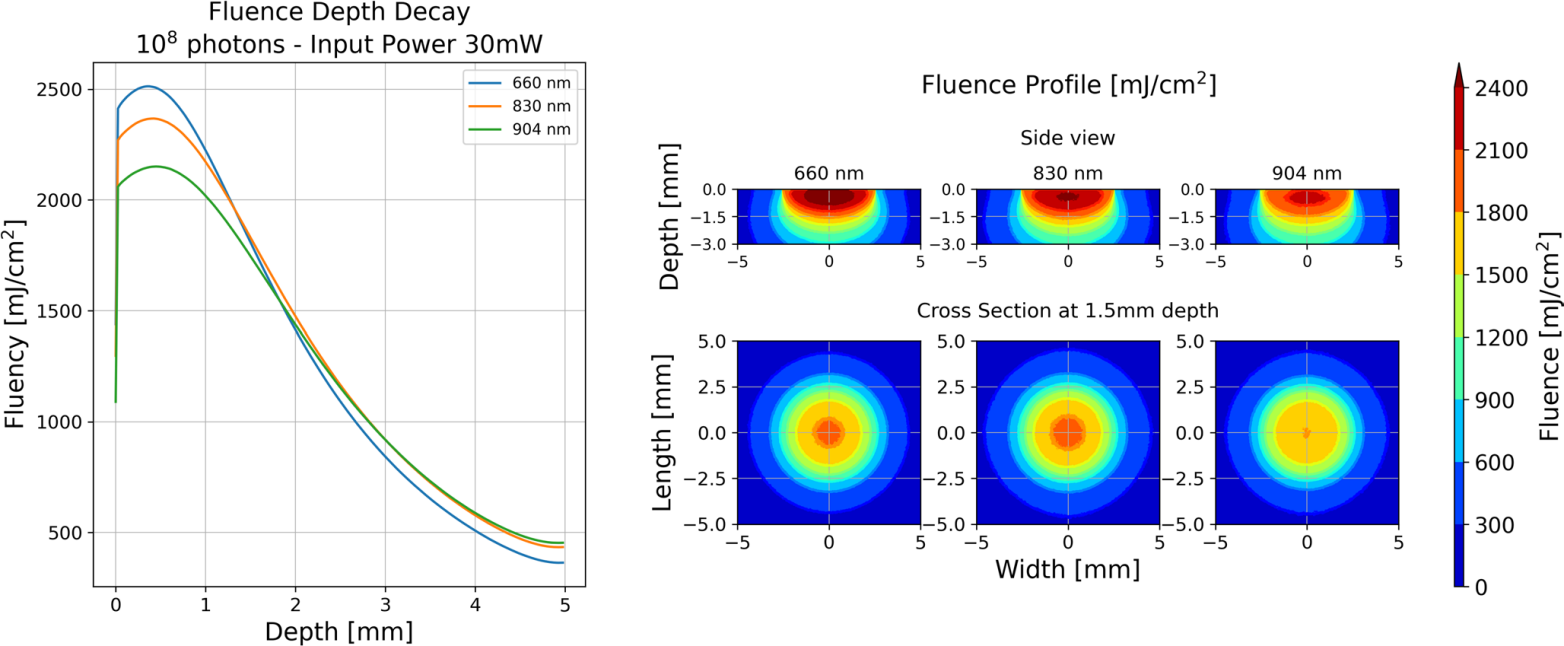
Fluence profile in simulations of different wavelengths

Figure 1 illustrates the internal fluence rate distribution within the skin model for wavelengths of 660 nm, 830 nm, and 904 nm. Simulations were performed using a 30 mW input power, 1 s exposure time, and a perfectly collimated circular beam with 1 mm radius to ensure standardized comparison across spectral conditions.

At the tissue surface (z = 0 mm), the 660 nm condition presented the highest peak fluence rate among the three wavelengths. In all simulations, the maximum fluence was observed slightly below the tissue surface rather than exactly at the boundary. This behavior reflects the well-established fluence build-up effect in turbid media, resulting from forward-directed scattering and boundary reflection losses at the air–tissue interface. The 830 nm and 904 nm simulations showed slightly lower surface peak values under identical power and geometric conditions. This small difference likely reflects wavelength-dependent variations in the optical coefficients used in the simulations, particularly scattering-related effects influencing near-surface energy deposition.

All wavelengths exhibited exponential attenuation with increasing depth. At 1 mm depth, the fluence rate decreased to approximately 5% of the respective surface value for all three wavelengths. The attenuation profiles remained closely aligned throughout the first 2 mm of tissue, with no marked divergence in axial decay behavior under the simulated conditions.

Two-dimensional fluence rate maps further demonstrated symmetrical energy distribution centered along the beam axis. Sagittal cross-sections revealed comparable axial penetration patterns across wavelengths, while transverse sections at 1.5 mm depth showed concentric fluence contours with similar lateral spread. Minor differences in lateral confinement were observed between wavelengths; however, the overall spatial distribution pattern remained consistent under controlled beam geometry and power.

### Effect of beam divergence on fluence decay

**Fig. 2:**
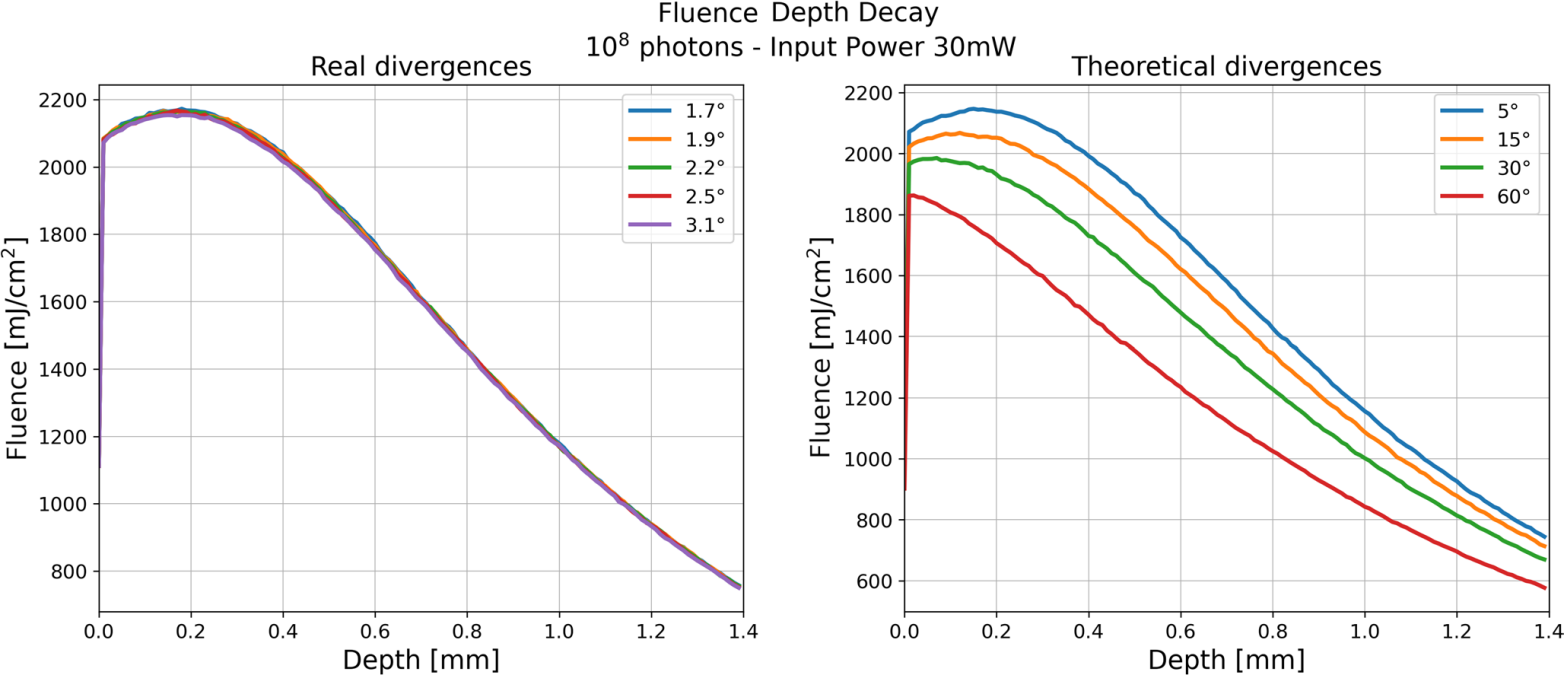
Fluence profile in simulations of different divergences, in real or theoretical settings. The values were simulated for the 904 nm. The real divergence values were obtained experimentally, whereas the theoretical values were extrapolated to investigate the influence of larger divergence angles

Figure 2 presents the axial fluence rate decay profiles obtained under different beam divergence conditions at 904 nm. All simulations were performed using a 1 mm radius circular beam, 30 mW input power, and 1 s exposure time to isolate the effect of angular spread.

For experimentally measured divergence values ranging from 1.7° to 3.1°, the axial fluence rate profiles were nearly superimposed. Across the first 2 mm of tissue depth, differences in fluence rate between these real-device divergence values were minimal, with comparable surface peak values and similar attenuation slopes. At 1 mm depth, fluence rate decreased to approximately 50% of the surface value under all real divergence conditions.

In contrast, simulations using larger theoretical divergence angles (5°, 15°, 30°, and 60°) demonstrated progressive modification of axial fluence rate behavior. Increasing divergence resulted in reduced surface peak fluence rate and steeper depth-dependent attenuation. The 60° divergence condition exhibited the most pronounced decay, reaching less than 40% of its surface value at 1 mm depth. Intermediate divergence values (15° and 30°) showed attenuation patterns between the collimated and 60° conditions.

Two-dimensional fluence rate maps revealed increasing lateral spread of energy distribution with larger divergence angles. While the collimated and low-divergence beams maintained concentrated axial energy profiles, high-divergence conditions produced broader transverse distributions accompanied by reduced central-axis fluence rate.

### Influence of source radius and real device parameters on fluence distribution

**Fig. 3:**
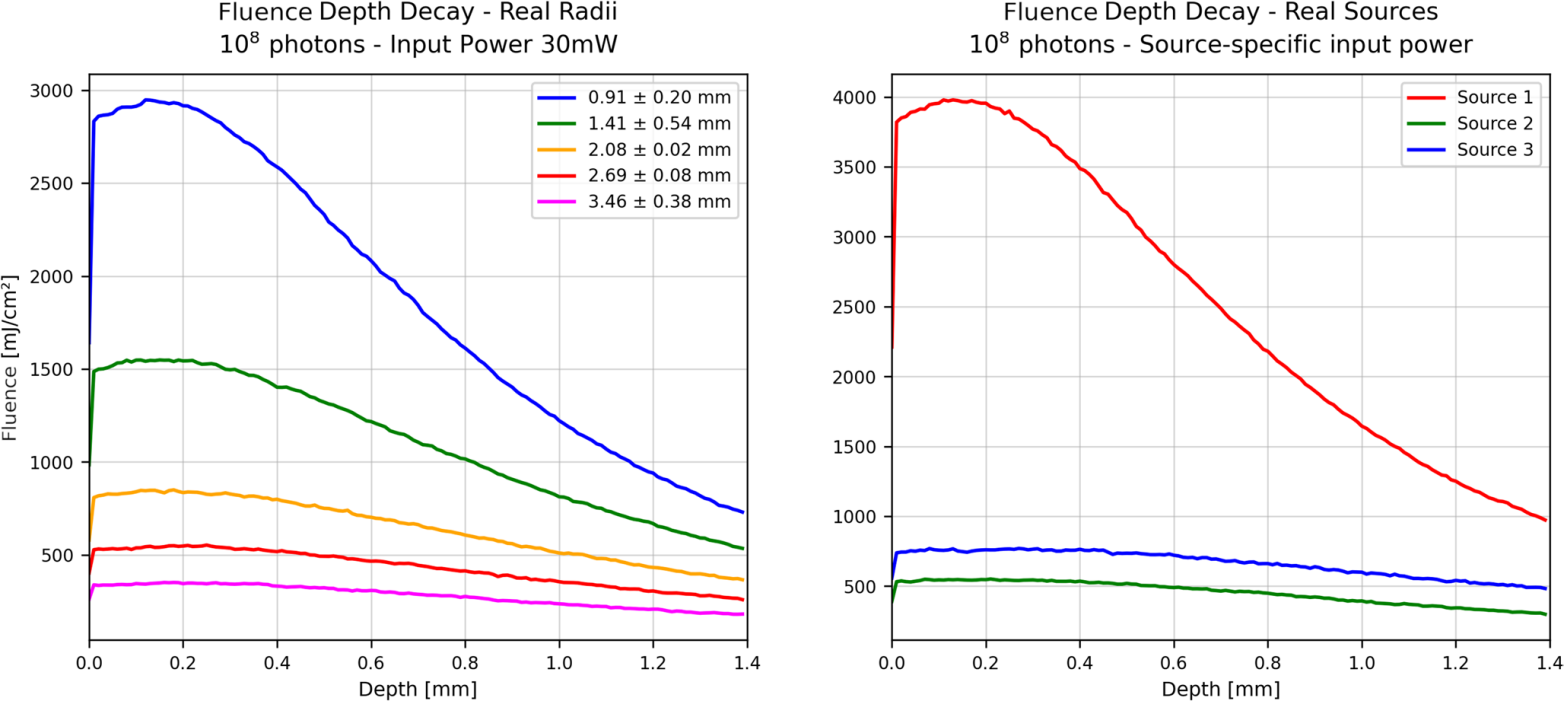
Fluence profile in simulations of different source radius and real sources parameters (Source 1 parameters: 660 nm, 40 mW, 0.9 ± 0.2 mm; Source 2 parameters: 830 nm, 30 mW, 2.7 ± 0.1 mm; Source 3 parameters: 904 nm, 70 mW, 3.5 ± 0.4 mm)

Figure 3a illustrates the axial fluence rate decay profiles obtained for different beam radii under constant total input power (30 mW) and 1 s exposure time. Five beam radii ranging from 0.91 mm to 3.46 mm were simulated to isolate the geometric effect of source size.

Under identical total power conditions, decreasing beam radius resulted in increased surface peak fluence rate. The smallest radius (0.91 mm) produced the highest entrance fluence rate, whereas larger radii generated progressively lower surface peaks due to energy distribution over a wider area. As beam radius increased, axial fluence rate profiles exhibited lower central-axis values throughout depth, while maintaining similar exponential attenuation behavior.

Although all conditions demonstrated depth-dependent decay, smaller radii maintained higher absolute fluence rate values along the first millimeters of tissue compared to broader beams. The differences in axial fluence rate magnitude were most pronounced at the tissue surface and remained observable up to 2 mm depth.

Figure 3b presents simulations using the experimentally measured parameters of three commercial laser sources. Despite differences in wavelength and output power, surface fluence rate magnitude was primarily influenced by beam radius. Source 1 (660 nm, 40 mW, 0.9 mm radius) exhibited the highest surface peak fluence rate. Source 2 (830 nm, 30 mW, 2.7 mm radius) showed intermediate values, while Source 3 (904 nm, 70 mW, 3.5 mm radius) presented lower peak fluence rate despite its higher nominal power.

Across all three devices, axial attenuation followed similar decay patterns; however, absolute fluence rate magnitude along the central axis remained dependent on the initial beam radius. These findings demonstrate that beam geometry substantially modulates internal fluence rate distribution under clinically relevant power settings.

### Fluence distribution by radius and real source properties

**Fig. 4:**
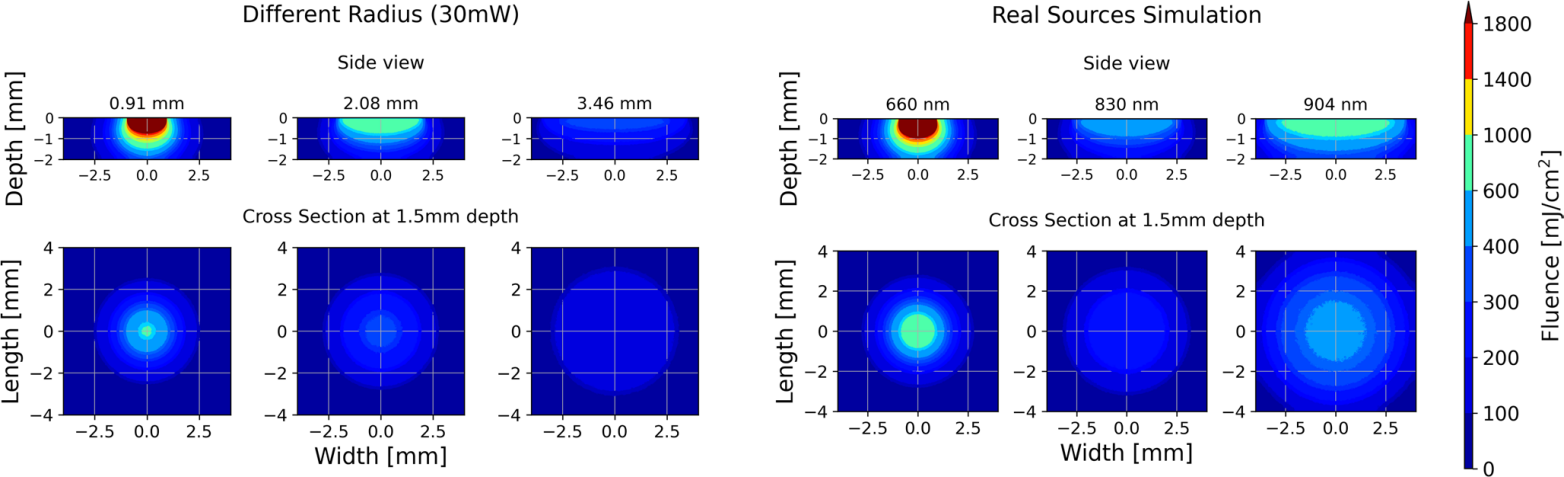
Spatial fluence distribution at 1.5 mm depth in skin phantom. Upper panels: Side (sagittal) and cross-sectional (transverse) views comparing three real diode lasers: Source 1 (660 nm, 40 mW, 0.9 mm radius – tightest contours), Source 2 (830 nm, 30 mW, 2.7 mm radius – intermediate), Source 3 (904 nm, 70 mW, 3.5 mm radius – widest spread). Lower panels: Isolated beam radius effect (660 nm, 30 mW fixed) showing 0.91 mm (highest central fluence) vs. 3.46 mm radius (broader, lower intensity). Contours represent fluence rate (W/cm²)

Figure 4 presents the spatial fluence rate distribution at 1.5 mm depth within the skin model, including sagittal (side) and transverse (cross-sectional) views under different simulation conditions.

The upper panels compare three commercially available laser sources with distinct wavelength, power, and beam radius parameters. Source 1 (660 nm, 40 mW, 0.9 mm radius) exhibited the highest central-axis fluence rate concentration at 1.5 mm depth, characterized by tightly clustered contour lines. Source 2 (830 nm, 30 mW, 2.7 mm radius) demonstrated a broader spatial distribution with moderate central fluence magnitude. Source 3 (904 nm, 70 mW, 3.5 mm radius) showed the widest transverse spread and lower central-axis peak fluence rate relative to Source 1, despite its higher nominal power.

The lower panels isolate the effect of beam radius while maintaining constant wavelength (660 nm) and input power (30 mW). Under these controlled conditions, decreasing beam radius resulted in greater central fluence rate concentration and steeper radial gradients. The 0.91 mm radius condition produced a compact high-fluence region centered along the beam axis, whereas the 3.46 mm radius condition generated a wider distribution with reduced peak magnitude.

Across all simulations, the spatial fluence rate pattern remained radially symmetric under collimated conditions, with contour expansion directly proportional to beam radius. Differences between conditions were primarily observed in central-axis magnitude and radial spread, rather than in overall distribution geometry.

## Discussion

This study employed Monte Carlo simulations incorporating experimentally measured laser parameters to characterize internal fluence rate distribution in human skin at 660 nm, 830 nm, and 904 nm. By integrating realistic beam geometry and divergence values, the simulations provide quantitative insight into subsurface optical energy deposition under clinically relevant conditions [9, 17, 18].

Within the therapeutic window (650–950 nm), light propagation in skin is governed primarily by scattering rather than absorption, resulting in exponential attenuation patterns across all simulated wavelengths [4, 19]. Although surface peak fluence rate varied slightly among wavelengths, axial attenuation profiles were comparable within the first millimeters of tissue. The 904 nm condition exhibited modestly greater lateral confinement at depth, consistent with the reduced scattering coefficients measured at longer wavelengths.

Among all investigated parameters, beam radius exerted the most pronounced influence on internal fluence rate magnitude. Under constant total power, reducing beam radius increased surface peak fluence rate and maintained higher central-axis values throughout the superficial tissue layers. Conversely, increasing beam radius distributed the same optical power over a broader entrance area, reducing peak fluence rate despite similar attenuation slopes [5]. These findings quantitatively demonstrate that geometric beam parameters may modulate subsurface fluence more substantially than nominal power differences within the investigated clinical range.

Beam divergence analysis further clarified that real-world divergence values (0.08–0.11 rad) minimally affected axial fluence rate within superficial depths under contact-mode conditions. However, larger angular spreads produced marked reductions in central-axis fluence retention and increased lateral energy dispersion. This suggests that beam collimation becomes increasingly relevant when broader divergence angles are present, such as in non-collimated or LED-based systems.

Compared with prior Monte Carlo studies employing idealized collimated beams or assumed source geometries [5, 9, 10], the present simulations incorporated experimentally measured beam radius and divergence parameters. This approach revealed discrepancies between nominal device specifications and effective subsurface fluence rate distribution, reinforcing the importance of detailed optical source characterization in dosimetric modeling.

Clinically, these findings indicate that beam geometry should be considered alongside wavelength and output power when defining irradiation parameters. Devices with smaller beam radii may generate higher superficial fluence rate, whereas larger beams promote broader spatial distribution with lower peak intensity. Consequently, dosimetric optimization requires integration of geometric, spectral, and power-related parameters rather than reliance on nominal power alone.

Limitations should be acknowledged. The optical properties used in the simulations were derived from Caucasian abdominal skin samples and therefore do not account for variations in melanin concentration, vascular perfusion, or anatomical site. This consideration may be particularly relevant at 660 nm, where melanin-dependent absorption differences may substantially alter superficial energy deposition, as suggested by previous experimental findings [6–8, 20]. Additionally, the model assumed a homogeneous tissue representation and static optical coefficients, which was intentionally adopted to isolate the effects of experimentally measured source parameters under controlled conditions. Although this simplification does not capture interface effects and layer-specific optical heterogeneity, it enables analysis of relative trends associated with beam geometry and divergence. Furthermore, the simulated fluence distributions were not directly validated against experimental measurements. Although Monte Carlo methods have been extensively validated for modeling light transport in biological tissues, future studies should incorporate anatomically realistic multilayer models and correlate simulations with in vivo or phantom-based measurements, particularly for absolute fluence quantification.

Overall, the present study demonstrates that Monte Carlo simulations incorporating experimentally measured laser parameters provide a robust framework for quantitative fluence rate prediction in biological tissues. Beam radius emerged as a dominant determinant of subsurface fluence magnitude within clinically relevant ranges, underscoring the need for standardized optical source characterization in photobiomodulation protocols.

## Conclusion

Among the investigated variables, beam radius exerted the strongest influence on subsurface fluence magnitude under constant power, whereas wavelengths within the therapeutic window (660–904 nm) showed comparable attenuation behavior in superficial tissue. Realistic divergence values typical of commercial devices minimally affected axial fluence under contact-mode conditions, while larger angular spreads significantly reduced central-axis fluence retention. These findings highlight the importance of integrating experimentally determined geometric source parameters into computational dosimetry models to improve precision and standardization in light-based protocols. However, these results should be interpreted within the optical conditions simulated here and may shift in higher skin phototypes, particularly at shorter wavelengths such as 660 nm where melanin absorption may substantially alter superficial fluence distribution.

## Data Availability

No datasets were generated or analysed during the current study.

## Abbreviations

3D: three-dimensional
g: Anisotropy factor
IAD: Inverse Adding-Doubling
MC: Monte Carlo
MCX: Monte Carlo eXtreme
n: Refractive index
PBMT: Photobiomodulation therapy
PDT: Photodynamic therapy
μ_a_: Absorption coefficient
μ_s_: Scattering coefficient
